# Integrating cognitive-behavioral training with immersive virtual reality intervention in ADHD: a case report

**DOI:** 10.3389/fpsyt.2026.1888297

**Published:** 2026-07-20

**Authors:** Carmela Settimo, Doriana Cantale Aeo, Antonino Lombardo Facciale, Laura Turriziani, Emanuela Tripodi, Caterina Impallomeni, Angelo Quartarone, Rocco Salvatore Calabrò, Francesca Cucinotta

**Affiliations:** IRCCS Centro Neurolesi “Bonino-Pulejo”, Messina, Italy

**Keywords:** ADHD, case report, cognitive-behavioral therapy (CBT), immersive virtual reality (IVR), intervention, neurorehabilitation, pediatric

## Abstract

**Introduction:**

Attention-Deficit/Hyperactivity Disorder (ADHD) is a condition characterized by persistent patterns of inattention and/or hyperactivity-impulsivity. This case highlights the potential benefit of integrating Immersive Virtual Reality (IVR) with cognitive-behavioral therapy (CBT) in the rehabilitation of a child with ADHD. It contributes to emerging evidence by showing how a combined approach may simultaneously target executive, attentional, and motor domains within a single intervention.

**Case presentation:**

An 8-year-old child with deficits in sustained and selective attention, impaired executive functioning (including planning and working memory), impulsivity, and difficulties in motor regulation, as revealed during baseline assessments, impacting daily functioning. The patient was diagnosed with combined-type ADHD and underwent a 12-week CBT intervention, followed by integrated IVR-CBT intervention targeting executive functions and self-control, once a week for 12 weeks. The intervention was conducted using the CAREN (Computer Assisted Rehabilitation Environment), an immersive virtual reality platform integrating multisensory input and interactive tasks to promote cognitive and motor engagement. Post-intervention assessments showed improvements in sustained and selective attention, planning, working memory, and balance. There was also an increase in involvement and a reduction in impulsivity.

**Conclusion:**

The findings support the hypothesis that immersive, embodied interventions targeting both executive and sensorimotor processes may represent a promising novelty adjunctive rehabilitation approach. Further studies are needed to evaluate efficacy, generalizability, and to confirm these findings in larger samples. This case report was prepared in accordance with the CARE Guidelines.

## 1 Introduction

Attention-Deficit/Hyperactivity Disorder (ADHD) is among the most relevant neurodevelopmental disorders, with global prevalence estimates ranging from 5% to 7% in children and adolescents (1, 2). The condition is characterized by persistent patterns of inattention and/or hyperactivity–impulsivity and its multifactorial etiology involve neurobiological, genetic, and environmental determinants (3).

Several impairment have been observed across a wide range of neurocognitive domains (4). In particular, ADHD symptoms affect executive functions (5), and can have a substantial impact on a child’s daily functioning, significantly affecting their quality of life and that of their family (6, 7).

From a clinical perspective, ADHD is associated with marked impairments across academic, social, and behavioral domains (8) and is frequently comorbid with other neurodevelopmental or psychiatric conditions, thereby contributing to highly complex and heterogeneous clinical profiles (9).

Although evidence-based interventions, particularly cognitive-behavioral therapy (CBT), have been shown to enhance self-regulatory processes and executive functioning (10, 11), conventional therapeutic approaches present several limitations. These include challenges in providing ecologically valid exposure, limited standardization of stimuli, and a reduced ability to establish highly controlled clinical contexts (12, 13). In contrast, virtual reality (VR) technologies address many of these constraints by enabling immersive, dynamically adjustable environments that can be precisely tailored to individual therapeutic needs, thereby enhancing treatment efficacy (14).

Several studies have explored the applicability and efficacy of VR interventions in pediatric populations. Non-immersive VR has been used in speech therapy for children with Developmental Language Disorders (15) and cerebral palsy (16), and immersive VR has been integrated into rehabilitation program for children with Global Developmental Delay (17). Recent evidence strongly supports the clinical value of VR-based interventions: Capobianco et al. (18) reported that VR applications in neurodevelopmental disorders offer high ecological validity and may improve attention, executive functioning, and socio-emotional skills. Similarly, Passaro et al. (19) emphasized that VR complements traditional interventions by facilitating personalized, multimodal therapeutic programs capable of enhancing adaptive behavior, motor control, and learning processes across heterogeneous neurodevelopmental profiles.

A growing body of literature supports the integration of VR within CBT, indicating that such approaches may achieve outcomes comparable to standard CBT while offering additional advantages in terms of ecological validity, patient engagement, and the implementation of exposure−based and cognitive training interventions across diverse psychiatric and neurodevelopmental populations (20, 21).

Furthermore, immersive virtual reality (IVR)-based interventions has shown promise in improving attention, executive functions, and social skills in children, showing promising results compared to conventional therapies (20). Specifically, in the ADHD population, large effect sizes have been reported for attention and memory, although the methodological quality of studies remains limited (21) with scarse data on usability.

Among the most advanced solutions, the Immersive Virtual Reality platform Computer Assisted Rehabilitation Environment (CAREN; MOTEK Medical; Amsterdam, Netherlands) stands out for its ability to provide a highly interactive and customizable therapeutic setting tailored to individual patient needs (22). The platform integrates visual, auditory, vestibular, and tactile stimuli, allowing clinicians to introduce cognitive, visual, and physical perturbations that require users to respond dynamically during gait tasks (23).

Over time, CAREN has also been used to assess gait in children, enabling the study of gait characteristics in children with spastic cerebral palsy during inclined treadmill walking in a virtual reality environment (24), as well as the development of a virtual-reality treadmill gait database at different speeds in typically developing children (25).

Multiple studies demonstrate CAREN’s effectiveness across various neurological conditions in improving both motor and cognitive abilities while maintaining high patient motivation (26–28).

Despite these promising findings, further research is needed to establish the effectiveness, feasibility, and safety of IVR interventions in children. Specifically, to our knowledge, no studies have verified the usability and utility of CAREN system for ADHD population.

Accordingly, the present single-case study aims to describe the clinical use of an IVR technology-based setting using the CAREN system within a rehabilitation program for an 8-year-old ADHD child, focusing on observed cognitive and behavioural changes during the care episode.

## 2 Methods

This case report was prepared in accordance with the CARE Guidelines (29) to ensure transparent, complete, and high quality clinical case reporting. A single-case A-B sequential design was adopted. The patient first underwent a phase of standard CBT, followed by a second phase in which an IVR intervention was added on. Assessments were conducted at three time points: T0 (baseline), T1 (after CBT), T2 (after IVR + CBT), with an additional follow-up assessment conducted 3 months after.

This design allowed for the observation of changes over time and the potential additive contribution of the IVR-based intervention.

### 2.1 Case description

Pietro, fictitious name, is an 8-year-old boy with a diagnosis of ADHD, combined presentation, of moderate severity, accordingly with DSM 5 criteria (30). Family history of mood disorders and autism spectrum disorders was reported by parents. Genetic testing (karyotyping and CGH array) was performed and yielded negative results. Early psychomotor milestones achieved appropriately for age. From the age of three, significant hyperactivity and limited adherence to rules were observed. Parents described pronounced difficulties in behavioral self-regulation and impulse control, with attention deficits and difficulties in organizing activities. These behavioural patterns were reported across multiple daily contexts, often accompanied by reduced safety awareness and considerable difficulty in adult management. In contrast, his acquisition of personal autonomy skills progressed in line with age expectations. At age of 6, in light of the clinical framework derived from specialist evaluations, psychometric assessment, and direct observation, a diagnosis of ADHD, combined presentation, of moderate severity at age of six years old was established, in accordance with the Diagnostic and Statistical Manual of Mental Disorders, Fifth Edition (30). During the clinical assessment, intellectual quotient was evaluated with Leiter-3 (31), with a global Intelligent Quotient of 80. Baseline assessments of executive functions were also conducted, and diagnostic questionnaires were collected from teachers and parents. A negative neurological examination was performed by an experienced pediatric neuropsychiatrist; during the unstructured behavioral observation, delivered by a psychologist, a hyperkinetic behavioral pattern with rapid shifts in activities was observed.

Following the diagnosis, therapeutic support started weekly sessions of CBT a specific parent training was delivered for both caregivers. Qualitative improvements were observed in attention span, behavioral regulation, and pragmatic communication.

### 2.2 Participant enrollment and timeline

Pietro and his parents were invited to take part in a rehabilitation program involving the use of an IVR-based therapeutic activity, delivered as a complement to ongoing conventional therapy, performed at the Child Neuropsychiatry service of the IRCCS Centro Neurolesi “Bonino Pulejo” in Messina, Italy.

This study was carried out in accordance with the Declaration of Helsinki;

Written informed consent was obtained from both parents, and the child also provided written assent.

**Figure 1:**
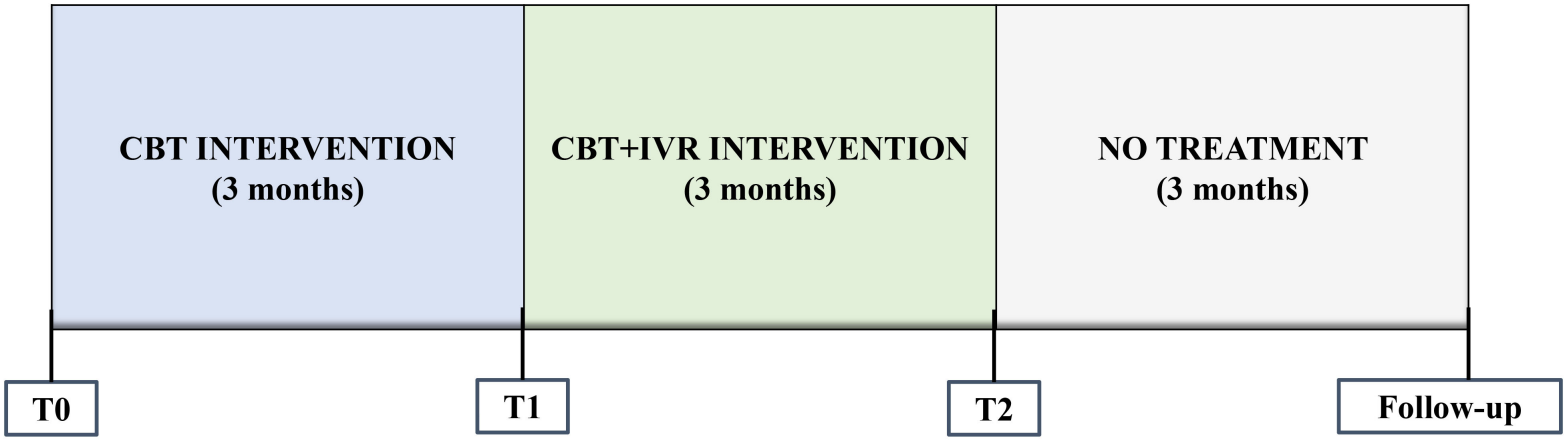
Timeline of the intervention.

The intervention consisted of a 3-month CBT phase followed by a 3-month combined IVR-CBT phase using in add-on a rehabilitation program with the use of CAREN system. Neuropsychological and behavioral assessments were conducted at baseline (T0), after CBT (T1), and after the combined intervention (T2), followed by a 3-month follow-up with repeated assessments (T3), detailed in **Figure 1**.

Following this, conventional rehabilitation is resumed with the same frequency and therapeutic objectives as before.

### 2.3 Assessment

A comprehensive neuropsychological assessment battery was administered at T0, T1, T2 and T3, to evaluate cognitive, executive, behavioral, and motor functioning, by a blind operator.

IVR safety and usability were evaluated using the Motion Sickness Assessment Questionnaire (MSAQ) (32) and the System Usability Scale (SUS) (33), respectively. Cognitive control and executive functions were assessed with the Stop-Signal Task (SST) (34) and the Tower of London (TOL) (35), together with the NEPSY–Second Edition (NEPSY−II) (36) to assess attention, executive functioning. Ecological executive, behavioral, attentional, and emotional functioning were measured through caregiver-reported instruments, including the BRIEF-2, Conners’ Parent Rating Scales–Third Edition (CPRS) (37), and the Child Behavior Checklist (CBCL) (38). Motor competence was assessed using the Movement Assessment Battery for Children–Second Edition (MABC-2) (39). In addition, both the child and the parents were asked to give their views on the results.

### 2.4 Therapeutic intervention

#### 2.4.1 Conventional therapy

In line with age-appropriate guidelines (9), the child received targeted individual CBT-intervention, consisted in weekly individual CBT-sessions, lasting one hour, to improve attention span, self-control, and impulse management skills.

In line with NICE guidelines (40), the intervention involved the use of structured and individually adapted psychological approaches embedded within a multimodal treatment framework, with outcomes targeting overall functioning rather than core symptoms alone.

The therapies were structured and developmentally tailored, integrating play-based tasks, visual aids, and guided practice to facilitate active participation and skill acquisition.

The child was supported in understanding the relations among thoughts, emotions, and behaviors, and how to identify early indicators of distractibility and impulsive tendencies. The intervention employed specific techniques, including task breakdown into manageable steps, the use of external cues to maintain attention, and the application of self-instructional strategies aimed at promoting response inhibition.

Contingency management procedures, such as positive reinforcement and immediate performance feedback, were systematically utilized to strengthen adaptive behaviors and increase task persistence. Additionally, simple coping strategies, such as deep breathing exercises and structured counting, were introduced to assist in regulating emotional arousal and reducing impulsive responses.

The intervention was delivered within a consistent and highly structured setting, with a gradual progression in task complexity to facilitate the generalization of acquired skills across academic and social environments.

#### 2.4.2 IVR-based treatment

**Figure 2:**
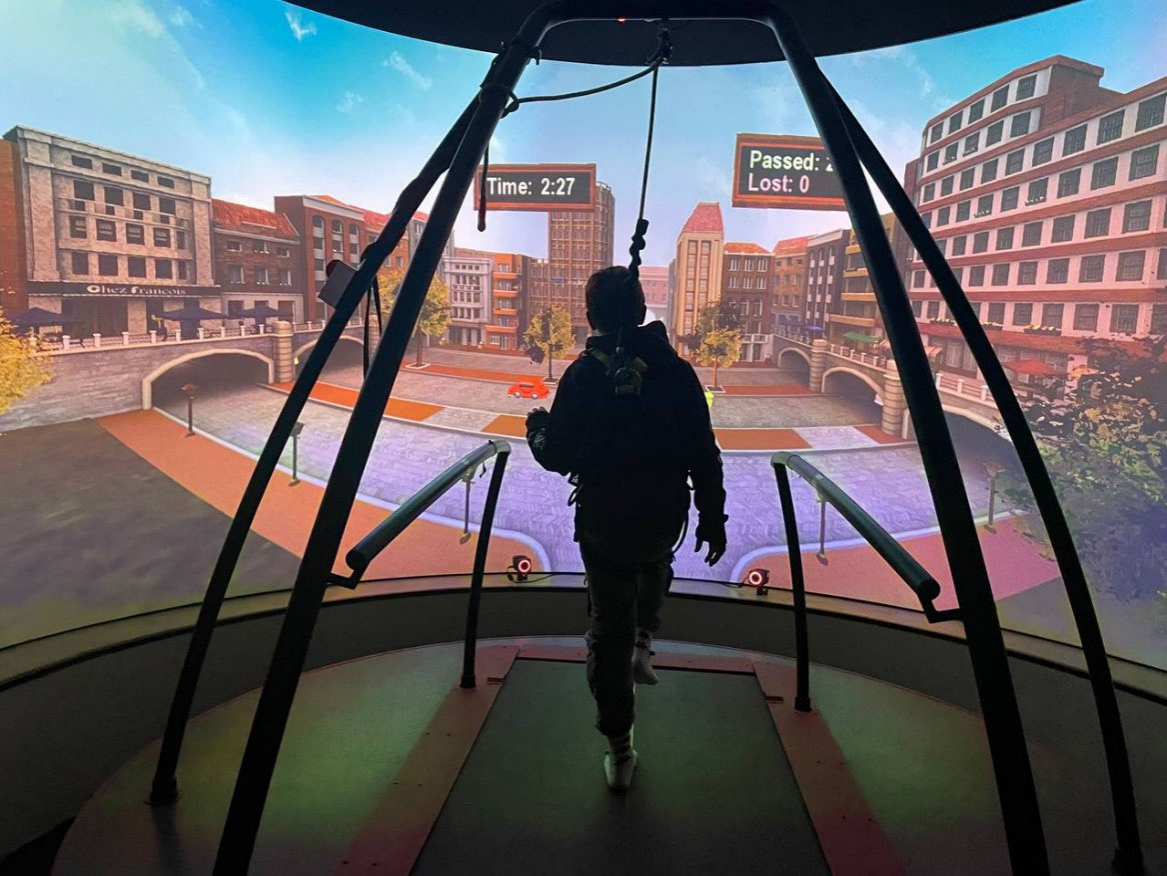
The child during the virtual reality session.

The protocol included a combination of conventional therapy (one hour per week) and immersive virtual reality–based therapy (one hour per week), to enhance attentional engagement and sustained focus by providing structured, multisensory and ecologically valid tasks. The IVR intervention was conducted using the CAREN system (Motek Medical, Amsterdam, The Netherlands), as in **Figure 2**.

The system integrates a motion capture setup with a six-degrees-of-freedom actuated motion base and an instrumented treadmill and supports different levels of VR immersion. The visuals are projected onto a 180-degree curved screen, with audio delivered through a surround sound system. During all sessions, the patient wore a full-body safety harness attached to an overhead support, ensuring safety without restricting movement. Markers are applied to specific points on the body according to the scenario to be performed, in order to enable the device to accurately capture movement.

Specifically, the program was designed to enhance sustained, selective, and divided attention through structured tasks. In parallel, the intervention aimed to strengthen executive functions, particularly response inhibition, cognitive flexibility, and goal-directed behavior, through the structured use of behavioral techniques, including contingent positive reinforcement, task-specific verbal prompting, and immediate performance feedback. These strategies were delivered in real time and tailored to the child’s responses to support response modulation, facilitate adaptation to progressively more demanding activities, and promote the development of self-regulation. This included fostering self-monitoring abilities, improving frustration management, and promoting the use of adaptive coping strategies during task performance, such as self-instruction (e.g., verbalizing task steps), brief response delay techniques to reduce impulsivity, and the use of simple problem-solving strategies (e.g., pausing, planning, and selecting alternative responses when errors occurred). Additional targets included the improvement of balance and adaptability under controlled sensory perturbations.

Our protocol used five virtual scenarios, detailed in **Table 1**. Each scenario was delivered for a fixed duration of three minutes and underwent two consecutive repetitions to enhance procedural consistency.

**Table 1:** Description of scenarios.

Scenario	Task description	Key interaction components	Skills trained
Rope Bridge	Crossing a suspension bridge with uphill and downhill segments while maintaining postural control.	Uneven terrain; moving gulls as distracting stimuli; inhibition of motor responses.	Dynamic balance; postural control; executive functions; goal-directed locomotion under cognitive load.
City Ride	Avoid incoming vehicles by shifting body weight laterally to control a virtual vehicle.	Lateral weight shift; dynamic visual stimuli; motor planning.	Motor planning; postural control; adaptive responses to dynamic stimuli.
Microbes	Avoid red viruses and capture green targets using whole-body movement; additional mode prevents virus entry via forward/backward movements.	Full-body movement across platform; adjustable difficulty and platform speed; avoidance and capture tasks.	Motor agility; coordination; reactivity; managing multiple task demands.
Traffic Jam	Avoid cars by lifting left/right leg depending on vehicle direction.	Continuous visual monitoring; selective limb activation; adjustable frequency and speed of vehicles.	Reaction time; selective motor responses; visual-motor coordination; anticipatory skills.
Boat	Navigate a boat avoiding buoys using trunk inclinations or center of pressure (CoP) shifts while platform simulates wave motion.	Trunk inclinations/CoP control; platform oscillations; target-oriented balance control.	Dynamic balance; trunk control; sensory integration; adaptation to perturbations.

## 3 Results

**Figure 3:**
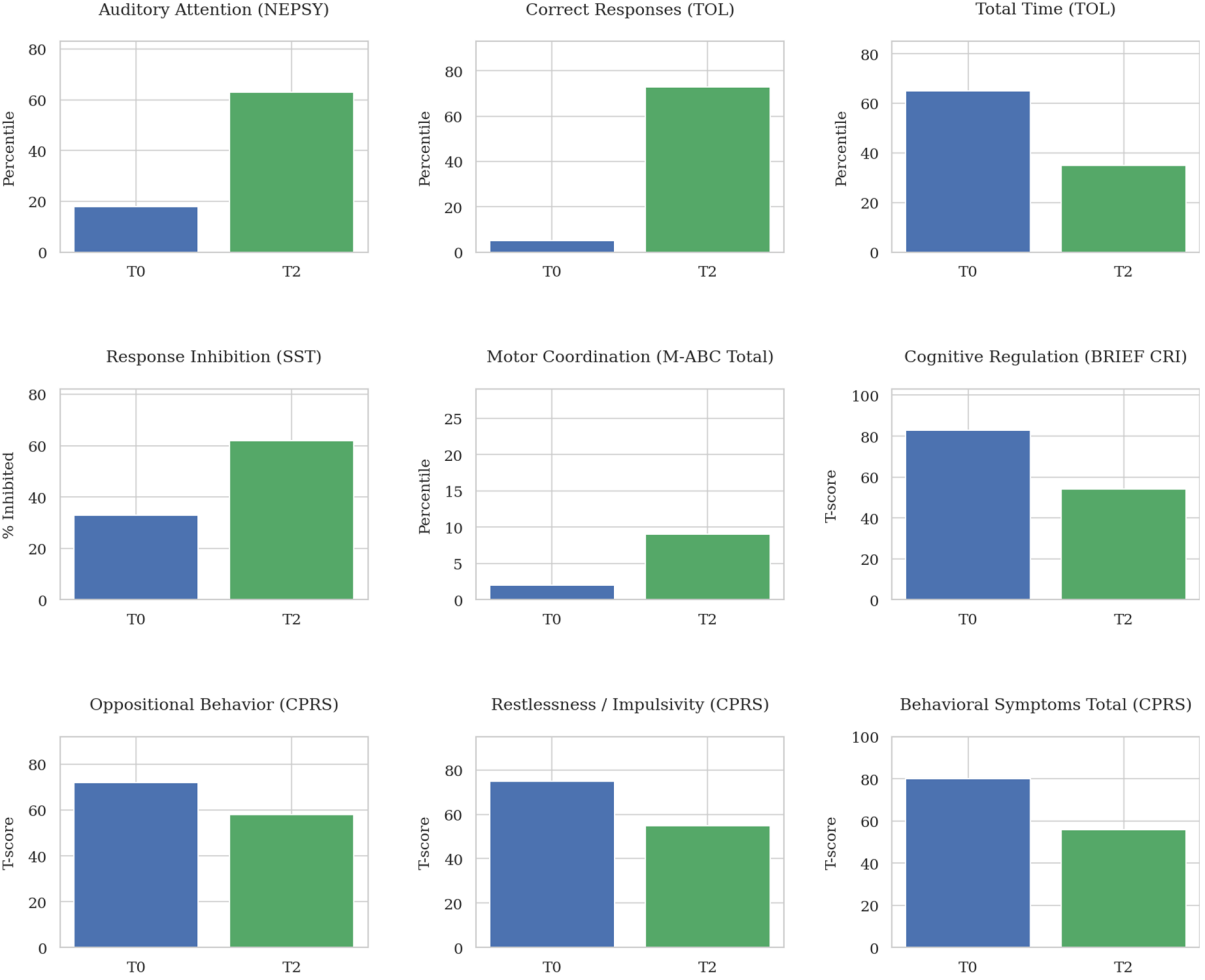
Longitudinal neuropsychological profiles across domains. Higher scores indicate better performance for percentile-based measures and inhibition rate, whereas lower T-scores reflect reduced executive and behavioral difficulties.

Across the three assessment points during treatment (T0, T1, T2) the child demonstrated a progressive improvement in attentional functioning, emotional–behavioral regulation, and executive control, detailed in **Figure 3**.

Performance on the NEPSY-II Auditory Attention subtest improved from the 11th–25th percentile at T0 to the 51st–75th percentile at T2, indicating a marked enhancement in sustained auditory attention. The TOL results shown an enhancement in planning and problem-solving processes over time. Specifically, total scores increased from the 5th percentile at T0 to the 70th–75th percentile at T2, while total task completion time decreased from the 65th percentile at T0 to the 35th percentile at T2, indicating greater efficiency in formulating and implementing goal-oriented strategies. However, the frequency of rule violations remained relatively high at all assessment points, suggesting persistent difficulties with inhibitory control and self-monitoring during the task. Inhibitory control, assessed using the SST, improved over time, with the percentage of correctly inhibited responses increasing from 33% at T0 to 62% at T2. The CBCL showed a clear reduction in symptom severity. Internalizing problems (e.g., anxiety, withdrawal) and externalizing behaviors (e.g., aggression, oppositionality) decreased significantly from T0 to T2, shifting from clinical or borderline ranges to values closer to normative functioning. Behavioral symptoms, assessed with CPRS, showed improvement in most domains. Oppositional behavior decreased from a T-score of 72 at T0 to 58 at T2, and restlessness/impulsivity decreased from 75 to 55. The total score improved from 80 at T0 to 56 at T2. Conversely, the ADHD Index increased from a T-score of 85 at T0 to 64 at T2. Everyday executive functioning, as measured by the BRIEF-2, showed a significant reduction in impairment. The Global Executive Composite (GEC) T-score decreased from 83 at T0 to 54 at T2, falling below the clinical cut-off (T ≥ 65). Motor skills, assessed using MABC-2, improved across all domains. Manual dexterity increased from the 1st percentile at T0 to the 5th percentile at T2; aiming and catching showed a marked improvement from the 5th percentile at T0 to the 63rd percentile at T2; balance improved from the 2nd percentile at T0 to the 16th percentile at T2. The total M-ABC score increased from the 2nd percentile at T0 to the 9th percentile at T2, indicating a global improvement in motor coordination.

Pietro did not report the occurrence of any symptoms typically associated with cybersickness.

Moreover, high usability was recorded, with no observed or reported operational difficulties during task execution; this was further supported by the SUS score of 85 (96th–100th percentile), reflecting an excellent level of system usability. Notably, high levels of treatment adherence were observed, with the child consistently participating in the sessions and demonstrating sustained engagement throughout the intervention period.

Progressive improvements were observed across attentional, executive, behavioral, and motor domains. Compared to baseline, the patient demonstrated enhanced planning efficiency, reduced task completion time, improved behavioral regulation, and decreased symptom severity on caregiver-reported measures. Notably, improvements in balance and motor coordination were also observed. The intervention was well tolerated, with no cybersickness reported and high treatment adherence.

The three-month follow-up assessment revealed results largely overlapping with those obtained at T2, indicating stability over time.

Motor performance remained stable across domains, with Manual Dexterity at the 5th percentile, Aiming and Catching at the 63rd percentile, and Balance at the 16th percentile. The overall MABC-2 Total Score remained within the 9th percentile, indicating persistence of motor difficulties but no evidence of regression following the completion of the intervention.

Performance on the Tower of London remained stable at the three-month follow-up (T3). Total scores were maintained within the 70th–75th percentile range, while total task completion time remained at the 35th percentile, indicating preservation of the planning and problem-solving gains achieved during treatment. Notably, the frequency of rule violations (n = 5) remained relatively unchanged across all assessment points, suggesting persistent difficulties in inhibitory control and self-monitoring. Performance on the NEPSY-II Auditory Attention subtest remained within the 51st–75th percentile range at T3.

Follow up data showed persistence of T2 results: inhibitory control (SST) remained 75% of responses correctly inhibited, as well as BRIEF-2 scores, GEC included. CBCL remained largely consistent with those observed at T2, indicating maintenance of the improvements achieved during the intervention. CPRS scores remained largely stable relative to post-treatment assessment. The transition from clinical-range scores at baseline to subclinical or non-clinical levels observed at T2 was maintained over time. Although slight variations were noted in some domains at follow-up, these changes did not exceed clinical cut-off values, indicating sustained improvement and no evidence of clinically significant relapse.

These findings support the durability of the improvements achieved during treatment and suggest that the benefits observed at post-intervention were maintained at the three-months.

### 3.1 Patient perspective

The child reported that the VR sessions were experienced as highly enjoyable and conducive to maintaining sustained attentional engagement. The immersive environments were perceived as more stimulating and less frustrating than conventional task-based activities.

Parents noted clear improvements in the child’s self-confidence, emotional regulation, and willingness to engage in homework related tasks. They also expressed strong satisfaction with the integrated IVR-CBT intervention, emphasizing that the child demonstrated a meaningful sense of achievement throughout the therapeutic activities.

## 4 Discussion

The present case report suggests that integrating CAREN-based IVR with conventional CBT may provide a meaningful enhancement of rehabilitation outcomes in ADHD children.

Despite the growing body of empirical research examining alternative VR-based intervention platforms, to the best of our knowledge, no previous studies have employed the specific CAREN IVR system specifically in this population.

Our study highlighted the good usability of the CAREN system, as reflected by the high level of patient engagement throughout the intervention. The immersive gaming component proved particularly effective in increasing engagement and motivation, which may be considered crucial elements in rehabilitation programs for ADHD, as previously reported in the literature on exergaming and VR-based cognitive training (41, 42). Interactive and dynamic virtual environments foster active participation and treatment adherence, helping to overcome some of the limitations of traditional rehabilitation approaches. In this sense, engagement may represent not only a facilitating factor, but also an active mechanism that may support cognitive and behavioral change (43).

Regarding safety, the intervention was well tolerated and did not result in adverse events. Although safety outcomes in VR interventions are still reported in a relatively limited number of studies, particularly in pediatric and neuropsychiatric populations, the available evidence consistently indicates a low risk profile. This aspect nevertheless represents an important area for further investigation, so that it may be better understood, given the specificity of both the intervention and the clinical population, as emphasized in recent systematic reviews (21, 44). The present findings contribute to this emerging field by providing preliminary evidence, within a single-case framework, supporting the feasibility and tolerability of immersive VR protocols in a child with ADHD. However, future research including larger samples is required to confirm these observations and to determine their generalizability across clinical populations.

Furthermore, the absence of a washout period constitutes a methodological limitation; however, it was not deemed appropriate to interrupt an ongoing CBT intervention in a pediatric clinical setting, given the priority placed on continuity of care and the patient’s well-being (47). This limits the ability to distinguish the specific contribution of the IVR component, and the observed improvements should therefore be interpreted as potentially reflecting cumulative treatment effects.

From a cognitive perspective, the most notable improvement was observed in auditory attention. Following the intervention, the patient demonstrated enhanced ability to monitor and respond to auditory stimuli in the standardized tests administered. This might suggest a strengthening of sustained and selective attention processes, that may have been potentially facilitated by the multisensory and interactive nature of the VR setting. Such an interpretation is consistent with prior findings indicating that immersive environments may effectively engage attentional mechanisms (45, 46). Over time, we also observed an enhancement in planning and problem-solving processes, in line with evidence suggesting that VR-based training may promote higher-order executive functions, including strategic planning (42, 44). Repeated exposure to complex tasks requiring anticipation, adaptation, and error monitoring may facilitate progressive refinement of these abilities.

In parallel, the patient demonstrated greater efficiency in formulating and implementing goal-oriented strategies, as also reported in studies involving immersive VR protocols that emphasize problem solving in dynamic, action-oriented environments (27, 46). This finding might indicate not only an improvement in isolated executive components but also that there may be an improvement in their functional integration within goal-directed behavior.

Results related to inhibitory control were mixed, consistent with previous findings (47–49). However, it is noteworthy that everyday executive functioning showed a significant reduction in impairment. This apparent discrepancy may be interpreted as improvements being more evident at a functional level than in isolated neuropsychological tests. In other words, the patient may have developed compensatory strategies that may allow more effective management of real-life demands despite persistent difficulties in specific executive domains. This discrepancy highlights the well-known gap between performance-based measures and ecologically valid functioning (44, 50, 51).

Another relevant outcome was the significant reduction in internalizing problems and externalizing behaviors from T0 to T2. This improvement may be interpreted within a broader self-regulation framework. The structured and predictable nature of the virtual environment, combined with immediate feedback and clear contingencies, may have supported the development of more adaptive regulatory strategies (52). Additionally, increased engagement and repeated experiences of success may have enhanced self-efficacy, thereby reducing frustration, emotional dysregulation, and behavioral reactivity. Although this interpretation should be considered with caution, it is consistent with the emerging literature on VR interventions in neurodevelopmental disorders (44). Moreover, these changes may also hypothetically reflect improved top-down regulatory control, which is typically impaired in ADHD and closely linked to both emotional dysregulation and behavioral difficulties (53).

Indeed, despite the persistence of core ADHD symptoms, psychometric assessments indicated that the patient might acquire new compensatory strategies that enhanced functional performance in daily activities. This finding supports the hypothesis that VR interventions may promote adaptive functioning and strategy use rather than complete symptom remission, so that functional gains may be achieved in line with neuroadaptive plasticity models (26). From this perspective, the goal of intervention shifts from symptom reduction to functional optimization, so that overall functioning may be improved.

This study has several limitations. Firstly, it is a single-case report, and secondly, the study design does not allow for clear attribution of effects to the IVR intervention alone. We acknowledge that findings from a single case report have many limitations, including epidemiological bias, impossibility of causal inference and generalization and over-interpretation. Thus, our results should be confirmed by well-designed clinical trials, taking into account long-term effects of this novel training.

However, the global enhancement of executive functions observed in this study is consistent with multiple lines of evidence supporting the efficacy of immersive VR protocols, and specifically with available data on the usability, safety, and usefulness of the CAREN system. Studies conducted across diverse clinical populations, including cerebellar ataxia, Parkinson’s disease, stroke, traumatic brain injury, and multiple sclerosis, have reported similar improvements in attention, executive functions, and motor performance (23, 26–28, 54, 55).

This convergence suggests that CAREN’s effectiveness maynot be strictly diagnosis-specific but may instead relate to its ability to stimulate core neurocognitive processes, such as immersive engagement, multisensory integration, and real-time feedback. Accordingly, the findings of the present case support the hypothesis that this immersive VR system may represent a flexible and promising rehabilitation tool, with potential applicability to ADHD and beyond, so that broader clinical applications may be explored.

Nevertheless, although preliminary, this report provides initial evidence supporting the feasibility, safety, and potential added value of CAREN-based interventions in ADHD, highlighting a novel rehabilitation approach that warrants further investigation.

## 5 Conclusion

Although these observations are based on a single case, the findings cautiously suggest that VR intervention provided with CAREN system could be a valuable addition to ADHD intervention, In this single case, the intervention was associated with potential improvements in functional outcomes, alongside enhanced engagement, motivation, and adherence to the rehabilitation program. Further investigations are needed to more robustly assess its feasibility and potential effectiveness.

The findings may support the hypothesis that immersive, embodied interventions targeting both executive and sensorimotor processes may represent a promising adjunctive rehabilitation approach. However, these observations are inherently limited by the single-case nature of the study and should therefore be interpreted with caution. Further studies are needed to evaluate generalizability and long-term stability of results.

## Data Availability

The raw data supporting the conclusions of this article will be made available by the authors, without undue reservation.
